# Development of novel EST microsatellite markers for genetic diversity analysis and correlation analysis of velvet antler growth characteristics in Sika deer

**DOI:** 10.1186/s41065-020-00137-x

**Published:** 2020-06-26

**Authors:** Boyin Jia, Guiwu Wang, Junjun Zheng, Wanyun Yang, Shuzhuo Chang, Jiali Zhang, Yuan Liu, Qining Li, Chenxia Ge, Guang Chen, Dongdong Liu, Fuhe Yang

**Affiliations:** 1 College of Animal Science and Technology, Jilin Agricultural University, 2888 Xincheng Street, Changchun, 130118 China; 2 Institute of Wild Economic Animals and Plants and State Key Laboratory for Molecular Biology of Special Economical Animals, Chinese Academy of Agricultural Sciences, 4899 Juye Street, Changchun, 130112 China; 3 College of Vocational and Technical Education, Changchun Sci-Tech University, 1699 Donghua Street, Changchun, 130606 China; 4 Key laboratory of Straw Biology and Utilization, The Ministry of Education, Jilin Agricultural University, 2888 Xincheng Street, Changchun, 130118 China; 5 College of Engineering and Technology, Jilin Agricultural University, 2888 Xincheng Street, Changchun, 130118 China

**Keywords:** EST microsatellite markers, Genetic diversity, Sika deer, Transcriptome, Velvet antler

## Abstract

**Background:**

Sika deer is one of the most popular and valued animals in China. However, few studies have been conducted on the microsatellite of Sika deer, which has hampered the progress of genetic selection breeding. To develop and characterize a set of microsatellites for Sika deer which provide helpful information for protection of Sika deer natural resources and effectively increase the yield and quantity of velvet antler.

**Results:**

We conducted a transcriptome survey of Sika deer using next-generation sequencing technology. One hundred eighty-two thousand two hundred ninety-five microsatellite markers were identified in the transcriptome, 170 of 200 loci were successfully amplified across panels of 140 individuals from Shuangyang Sika deer population. And 29 loci were found to be obvious polymorphic. Number of alleles is from 3 to 14. The expected heterozygosity ranged from 0.3087 to 0.7644. The observed heterozygosity ranged from 0 to 0.7698. The polymorphism information content values of those microsatellites varied ranged from 0.2602 to 0.7507. The marker-trait association was tested for 6 important and kernel characteristics of two-branched velvet antler in Shuangyang Sika deer through one-way analysis of variance. The results showed that marker-trait associations were identified with 8 different markers, especially M009 and M027.

**Conclusions:**

This study not only provided a large scale of microsatellites which were valuable for future genetic mapping and trait association in Sika deer, but also offers available information for molecular breeding in Sika deer.

## Background

Molecular markers are commonly used to test the genetic characteristics of species within or among populations [1]. These molecular markers mainly include microsatellite, single nucleotide polymorphisms (SNPs), random amplified polymorphic DNA (RAPD), amplified fragment length polymorphisms (AFLPs), sequence-related amplified polymorphisms (SRAPs) and so on [2]. Microsatellite markers are tandem repeats of 1–6 bp, usually associated with copy slippage and DNA repair, and are distributed in the genome of all organisms [3]. Microsatellite markers are characterized by high polymorphism, high information content, specificity, dominance, and reproducibility [4]. They can be classified into genomic microsatellite and expressed sequence tag microsatellite (EST microsatellite) based on their locations [5]. EST microsatellite was found in selectively more constrained regions of the genome [6]. Although compared with genomic microsatellite, EST microsatellite has some disadvantages: (1) The polymorphism level of EST microsatellite was lower than that of genomic microsatellite; (2) Due to a consequence of the undetected presence of introns in flanking regions, the amplicon size may be different from expected [7]. However, this was balanced by the following advantages over genomic microsatellite: (1) EST microsatellite was used to detect the variation in the expressed portion of the genome, which may give the concerned marker-trait associations; (2) EST microsatellite could be developed at no cost from the EST databases which become a fast, efficient and low-cost choice; (3) Unlike genomic microsatellite, once developed, EST microsatellite may be highly conserved among a number of related species, resulting in a high level of transferability [6, 8].

Due to the lack of available genomic information, it is difficult to develop ideal microsatellite. Transcriptome sequencing is an alternative to whole-genome sequencing for obtaining microsatellite, which are essential for developing abundant EST microsatellite and identifying target genomic regions for important characteristics [9]. In the last decade, deep transcriptome sequencing has been generated in model and non-model organisms, which have greatly accelerated the collection of genetic resources and the understanding of the interest of gene expression, regulation and networks [10–12]. Because on these advantages, transcriptome sequencing has been widely used in non-model animals for EST microsatellite development, including the Siberian tiger, the ridgetail white prawn *Exopalaemon carinicauda*, the Yellow catfish and so on [13–15].

Sika deer is one of the most popular and valued animals in China [16]. The velvet antler has long been a traditional tonic or alternative medicine, according to ancient Chinese pharmaceutical monographs [17]. It is also a useful model for the study of the mechanisms of organ regeneration, rapid growth and mineralization in mammals [18]. At present, the key problem of raising economic benefits of deer breeding is to improve the yield and quality of velvet antler in China. The growth characteristics of velvet antler are closely related to genetic factors. Despite substantial age- and environment-related variation, velvet antler size was also heritable [19]. Conventional breeding cycles are long, and it is difficult to make early selection. The research on the velvet antler growth characteristics by using molecular genetic markers can effectively overcome the shortcomings of traditional methods and improve the velvet antler yield of Sika deer. Due to the lack of sufficient molecular markers, Sika deer breeding technology is relatively backward. Although Zu et al. (2001) developed 200 microsatellite markers in Sika deer, most microsatellite markers were borrowed from cattle and the polymorphism was relatively low [20]. Until recently, 22,479 microsatellite markers were obtained from 125 M bp genomic data of Sika deer by using IlluminaHiSeq™2500 sequencing technology. Among them, 29 microsatellite markers were identified to be polymorphic, of which 10 loci could be used for paternity testing of Sika deer [21]. So far, only genomic microsatellite markers have been developed and EST microsatellite markers have not been reported. In addition, microsatellite markers have been mainly applied to genetic diversity, genetic structure analysis and paternity testing in Sika deer [22–24]. Only a few microsatellite markers related to the production performance of the velvet antler have been reported. Li et al. (2011) found that BE, CE genotype at BM1225 and AA genotype at T172 could be regarded as linkage genetic marker of velvet antler weight of Xingkaihu Sika deer [25]. Yang et al. (2014) found that the second exon C629G of melatonin receptor 1A gene showed significant association with velvet antler yield, and the velvet antler weight of CC genotype was higher than that of GC genotype in Wusan Sika deer [26]. However, complex characteristics could not be completely controlled by a single gene, and it required a series of genes.

To solve this problem, through Illumina sequencing and bioinformatics analysis, approximately 289 gigabytes (289G) high-quality reads were obtained and were assembled into 1,348,618 unigenes. In our study, the frequency and distribution of 182,295 potential EST microsatellite markers in the Sika deer transcriptome were analyzed. Two hundred EST microsatellite markers loci which expressed in antler were selected for primer design, of these, 170 loci were successfully amplified across panels of 140 individuals from Shuangyang Sika deer population. And 29 loci were found to be obvious polymorphic. Finally, the marker-trait association was tested for important and kernel characteristics of velvet antler in Shuangyang Sika deer. We found 8 EST microsatellites, especially M009 and M027, which can be used as molecular markers in the breeding process of weight of the two-branched velvet antler. The development of tremendous Sika deer molecular markers could help to protect germplasm resources. At the same time, it can effectively increase the yield and quantity of velvet antler. The present study thus represents the first comprehensive application of transcriptome sequencing in the development of Sika deer EST microsatellite markers.

## Results

### Characterization of EST microsatellites in the Sika deer Transcriptome

A total of 1,348,618 unigenes covering 0.74 GB and 182,295 EST microsatellite loci were identified from 182,295 sequences of unigenes. The average density of EST microsatellite was 9.53 kb. In total, 22,261 unigenes (12.21%) contained more than one EST microsatellite loci.

We analyzed the distribution of EST microsatellite that has 1–6 bp repeat motif. The mono-nucleotide was the most common type of repeat (70.15%), followed by di-nucleotide and tri-nucleotide (21.54, 7.4%), thirdly tetra-nucleotide, penta-nucleotide and hexa-nucleotide was only 0.91% of total EST microsatellite. In addition, the highest number of repeat motifs per locus was 11–36 (82,042, 45.01%). This was followed by 10 (47,829, 26.24%), 6 (23,194, 12.72%), 5 (10,764, 5.9%), 7 (10,132, 5.56%), 8 (4952, 2.72%) and 9 (3382, 1.86%) repeats (Table 1).

**Table 1:** Frequency of different repeat motifs among the EST microsatellite of Sika deer

Repeats	Mo	Di	Tri	Tetra	Penta	Hexa	Total	Percentage (%)
5	–	–	9244	1472	47	1	10,764	5.9
6	–	19,632	3438	118	5	1	23,194	12.72
7	–	9367	759	5	1	0	10,132	5.56
8	–	4904	42	4	1	1	4952	2.72
9	–	3375	3	1	2	1	3382	1.86
10	46,133	1696	0	0	0	0	47,829	26.24
11–36	81,754	284	0	3	0	1	82,042	45.01
Total	127,887	39,258	13,486	1603	56	5	182,295	100
Percentage (%)	70.15	21.54	7.4	0.88	0.03	2.74E-03	100	–

**Fig. 1:**
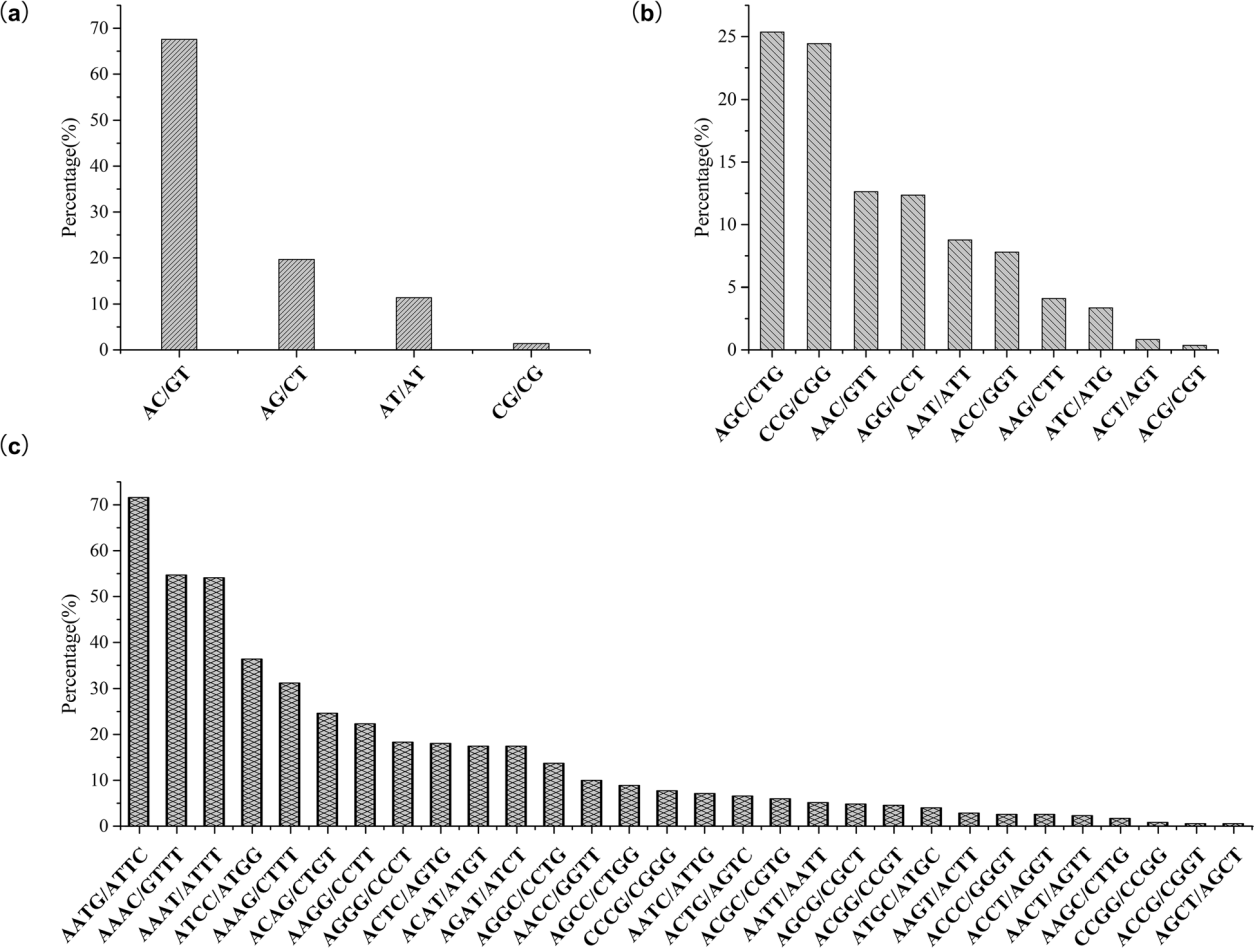
Frequency of different repeat motifs among the microsatellite markers of Sika deer was determined by transcriptome sequencing. **a** di-nucleotide repeats. **b** tri-nucleotide repeats. **c** tetra-nucleotide repeats

After the removal of the mono-nucleotide motifs, the di-nucleotide motifs were the main type in transcriptome of Sika deer (39,258, 72.15%) (Additional file 1). Among the di-nucleotide motifs, the most dominant type was AC/GT (26,522, 67.56%) (Fig. 1a). Two most frequent repeats in the tri-nucleotide were AGC/CTG (3420, 25.36%) and CCG/CGG (3296, 24.44%) (Fig. 1b). Among the tetra-nucleotide motifs, AATG/ATTC (250, 15.60%) was the most dominant motif (Fig. 1c).

In this study, the length of less than 12 bp microsatellite was removed. The total number of EST microsatellites screened from Sika deer was 72,316 (ranging in length from 12 bp to 313 bp). Among them, the lengths of 56,632 EST microsatellites (90.76%) were 12–20 bp, and the lengths of 6684 EST microsatellites (9.24%) were longer than 20 bp.

### Genetic diversity analysis

To verify the identified microsatellite markers, 18,692 primer pairs, which removed the length of less than 12 bp and the mono-nucleotide duplication microsatellites, were designed from 58,503 microsatellites expressed in velvet antler. Then, a total of 200 EST microsatellites of velvet antler were selected for validation. In this study, TP-M13 microsatellite PCR method was used to screen for primer. One hundred seventy of two hundred primer pairs successfully PCR-amplified, producing products of the expected size. Of the 170 primer pairs, 29 pairs showed polymorphisms among 140 shuangyang Sika deer samples (N_a_ ≥ 2). In total, 153 alleles were detected across the collected individuals, with the number of alleles per locus varying from 3 to 14. The average N_a_ was 5.6 and the average N_e_ was 2.6. The number of N_a_ of microsatellite M082 and M027 were 14 and 12 respectively. The number of N_e_ of microsatellite M005, M084, M099, M121, M131 and M132 were the least, all of which were 3 (Table 2).

**Table 2:** Characteristics of the 29 EST microsatellite markers and population genetic diversity analysis for Shuangyang Sika deer

M	Repeat Motif	Primer Sequences (5′–3′)	Tm	Size (bp)	Putative Function	N_a_	N_e_	H_e_	H_o_	PIC
M001	(TC)_10_	F: M13-TTCCCTTCTGGTTCTCAATCTC	60	246–252	Nop14-like family	4	3.1	0.6751	0.6496	0.6142
		R: CTGACTCTCACGTCTAAGCTCTTCT								
M002	(AC)_10_	F: M13-TATGAAAGGGCCTGGTTGTG	60	346–354	No significant match	4	1.1	0.3772	0.2791	0.2758
		R: CCACCATGTAGCCAGAATGG								
M005	(CT)_10_	F: M13-AATCGTATCACAGCACGGCT	60	282–288	No significant match	3	2	0.4983	0.518	0.4414
		R: CCCTGGTTAAGAGTCCCAGAT								
M009	(GA)_7_cag(GA)_10_	F: M13-CCACTAACTCTCCTGGCTGC	60	357–363	No significant match	4	2.4	0.5807	0.5333	0.5178
		R: AAGATGGTTTACTCAGTAATCGATG								
M027	(AT)_8_ttgaatccctctcctaaagcacagtatcttatataatactgtgattgcattga(TC)_9_	F: M13-TTCCTCACTTTCCTCATACATCTG	60	251–321	No significant match	12	2.1	0.5215	0.375	0.5029
		R: ATCTTCCAGTCAGGTGTTCAGC								
M037	(AC)_9_ccc(CA)_8_	F: M13-TCCCTCTAGCTGGAGCAATC	60	165–187	*Bos taurus* cytochrome P450 2C21-like	9	4.6	0.7845	0.6547	0.7507
		R: CTCTTGACAGTTTGGCATGTATAGT								
M073	(AT)_10_	F: M13-TTCAAATGAAGAAAGCTGAATG	60	109–113	*Capra hircus* family with sequence similarity 210	8	2.9	0.6546	0.5839	0.6031
		R: GTAAAAGTCACTTTTAAAGGGTCA								
M079	(CT)_10_	F: M13-CCTTAAAAGCATCATTTGAGCA	60	157–169	*Gorilla gorilla* gorilla transcription factor AP-2 alpha	5	2.8	0.649	0.6423	0.6083
		R: GCTATAACTGCTTCCAAAACGG								
M082	(AT)_10_	F: M13-CCATGTTTCTATTGGGTTGTCG	60	374–408	No significant match	14	3	0.6708	0.5547	0.6462
		R: AACTGGATCTACACTTCACACGGT								
M084	(GT)_10_	F: M13-AGGTCTTCCCCAGTGATGATG	60	422–424	No significant match	2	1.4	0.3087	0.292	0.2602
		R: GAGTCAATATCTGGAGCTCTGCC								
M089	(GA)_10_	F: M13-TTCCCTTCTGGTTCTCAATCTC	60	223–229	*Pantholops hodgsonii* thyroglobulin gene	4	3	0.6705	0.6475	0.6093
		R: CTCCTGTGTTGCTCACTCTCATC								
M092	(AC)_10_	F: M13-CCACCATCGGCGAAGAGTC	60	219–235	No significant match	5	2.7	0.6253	0.6331	0.5638
		R: TGCTGAGCCGACTTCCTGC								
M093	(TG)_10_	F: M13-TGTTGCTTGGGCTGTGGC	60	149–161	No significant match	6	2.1	0.532	0.4892	0.4623
		R: GTGGGCCTTAATCCAGCAAG								
M099	(AT)_10_	F: M13-ACAAATGGTGAAAACTCACTCC	60	202–208	Bos taurus probable E3 ubiquitin-protein ligase MID2-like	3	2.7	0.6281	0	0.5475
		R: GGTTCCTTAGTATCCTTGGCAC								
M102	(AT)_10_	F: M13-GGCTGCCTCGCTCTGTTCT	60	249–259	No significant match	6	3.3	0.7033	0.6763	0.6518
		R: GCGATGGATGAACATTCAAGAA								
M110	(GT)_10_	F: M13-GTTGGCTCTTGGTCCCTGAAT	60	155–177	*Homo sapiens* chromosome 5 clone CTD-2410 N18	9	3.4	0.7127	0.6449	0.665
		R: ATCCTCCTCCAGATTCTCCACTT								
M113	(GA)_10_	F: M13-AGGTTCCATCCTTGCCCTG	60	313–329	No significant match	6	2.9	0.6549	0.6544	0.605
		R: AACTTAGCCCAGTGAAATGCG								
M114	(AT)_10_	F: M13-GCTGGGTGACAAAGTGCTATG	60	166–176	Capra hircus family with sequence similarity 210	5	2.8	0.6477	0.6187	0.5962
		R: TGAAGAAAGCTGAATGAAAGTTG								
M117	(AC)_10_	F: M13-CACTGCATGCCTGCTTCCT	60	193–207	Pantholops hodgsonii snail family zinc finger 2	4	2.1	–	–	–
		R: TTTACTTGTCACTCCGCCTCTG								
M121	(CT)_10_	F: M13-GATCTCCCAGCCTGTCCTATG	60	251–255	No significant match	3	1.5	0.3413	0.3165	0.2912
		R: ACACTTCCCTGAGCTGACCTTAT								
M131	(AG)_10_	F: M13-CGAGTTCTGCTCATTGATGTG	60	164–172	*Ovis aries* Rho GTPase activating protein 19	3	2.3	0.5758	0.5827	0.4829
		R: TCCTGTGACTCAACTGATACCTC								
M132	(CT)_10_	F: M13-GAGTTCTGCTCATTGATGTGCT	60	149–157	Ovis aries Rho GTPase activating protein 19	3	2.3	0.5758	0.5827	0.4829
		R: GATACCTCTAACCTCACTCAGCG								
M136	(CA)_10_	F: M13-GACTCTTGGTCACAGCAGTCAC	60	207–221	Bos taurus SET domain containing 1B	5	2.3	–	–	–
		R: ACTTCTGTACATAGGCTGCCAT								
M139	(AG)_10_	F: M13-GCAGGAGTGAAAGGCAGATG	60	177–187	Bos taurus synapse differentiation inducing 1-like	6	2.5	0.6036	0.6475	0.5435
		R: GGATTCTGCACTCAGTGGCT								
M148	(CT)_10_	F: M13-TGATGCGTTCCTCTGTCAGC	60	383–385	Capra hircus transmembrane protein 164	4	2.1	0.5198	0	0.4682
		R: AATGGTCTTCCACTAGCCGC								
M157	(TC)_10_	F: M13-TTCTGACTGAGGAAGCGTCC	60	184–202	Homo sapiens chromosome 17, clone RP11-960B9	6	4.2	0.7644	0.7698	0.7265
		R: GGTCCCATGACTTAGCCTTAC								
M159	(CA)_10_	F: M13-TTCCAAACGCCAGAGGTAAC	60	207–215	*Bos mutus* ankyrin repeat domain 13A	5	3	0.6692	0.6763	0.6059
		R: GCTAGTTGAAGGATATGCAGGC								
M174	(AG)_10_	F: M13-CTTGTTGGAGGATGGATGGTT	60	120–132	Bos taurus protein inhibitor of activated STAT	4	2.3	0.5636	0.5683	0.4974
		R: TCCAAGGACAAGTGTTAGAAAGG								
M176	(AG)_10_	F: M13-GACACCACTTCTTGCCTCAAT	60	279–297	Bos taurus filamin B, beta	10	3.8	0.7391	0.252	0.6947
		R: ATTAAGCCACTTGTCTCTACAGG								
Average	–	–		–	–	5.6± 2.8	2.6± 0.8	0.6018 ± 0.1190	0.5127 ± 0.2010	0.5450 ± 0.1262

Among the 29 EST microsatellite loci, the H_e_ varied from 0.3087 to 0.7644, with an average of 0.6018 ± 0.1190, while the H_o_ varied from 0 to 0.7698, with an average of 0.5127 ± 0.2010. The PIC values ranged from 0.2602 to 0.7507, with an average of 0.5450 ± 0.1262 (Table 2). These 29 microsatellites exhibited medium and high levels of PIC (PIC > 0.25). The effective candidate microsatellite markers developed in this study would greatly promote the genetic diversity studies of varieties in Sika deer.

### Analysis of correlation between EST microsatellite and velvet antler characteristics

Characteristics of the model and coefficients that resulted from the multiple linear regressions were detailed in Table 3. The model suggested that main beam circle, main beam length, mouth circle, first tine circle, mouth length explain 86.9% of the normalized velvet antler weight (R^2^ = 0.869). Main beam circle (ß = 0.071, *P* < 0.001), main beam length (ß = 0.027, *P* < 0.001), mouth circle (ß = 0.043, *P* < 0.001), mouth length (ß = 0.016, *P* < 0.05), first tine circle (ß = 0.019, *P* < 0.05) were positively correlated with velvet antler weight. The correlation between EST microsatellite markers and the above growth characteristics of velvet antler of Shuangyang Sika deer was analyzed by one-way analysis of variance. The results showed that 8 EST microsatellite loci (M001, M009, M027, M084, M093, M113, M136, and M159) were significantly correlated with the above characteristics (Table 4).

**Table 3:** Multiple linear regression analysis of influencing factors and velvet antler weight

Independent Variable	ß value	Standard error	t value	*P* value	Model Parameters
Constant	−1.745	0.094	−18.659	0.000	R^2^ = 0.874 R^2^ adjusted = 0.869 F = 6.263 *P* = 0.014
Main Beam Circle	0.071	0.009	7.964	0.000	
Main Beam Length	0.027	0.006	4.414	0.000	
Mouth Circle	0.043	0.007	6.069	0.000	
Mouth Length	0.016	0.007	2.503	0.014	
First Tine Circle	0.019	0.007	2.753	0.007

**Table 4:** Correlation analysis between microsatellite loci polymorphism and velvet antler traits of Sika deer

Velvet Antler Trait	EST microsatellite	*P*-Value	R^2^
Velvet Antler Weight	M009	0.0403	0.147849
Velvet Antler Weight	M027	0.002742	0.338763
Main Beam Length	M009	0.021365	0.163189
Main Beam length	M084	0.008272	0.087284
Main Beam Length	M159	0.030037	0.165064
Main Beam Circle	M027	0.004684	0.326376
Main Beam Circle	M093	0.013715	0.183563
Mouth Length	M084	0.017309	0.074358
Mouth Circle	M001	0.031749	0.152333
Mouth Circle	M027	0.001891	0.347053
Mouth Circle	M113	0.004284	0.252471
Mouth Circle	M136	0.000159	0.272411

Bonferroni correction for multiple testing was applied to the *P*-value

**Fig. 2:**
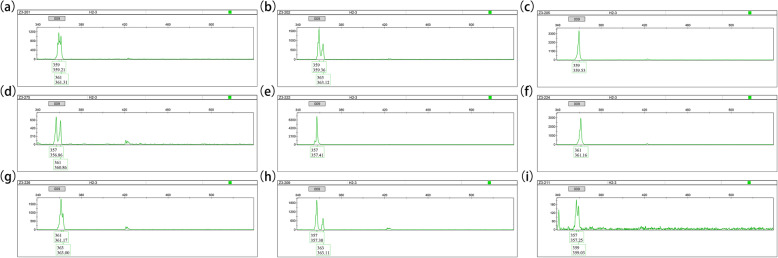
Typical fluorescence signals of the M009 microsatellite loci by capillary electrophoresis fragment analysis. The numbers above the sharp peaks represent length scales based on the internal size standard (bp). The numbers below the sharp peaks represent fragment lengths of PCR amplifications (bp). **a** 359/361, **b** 359/363, **c** 359/359, **d** 357/361, **e** 357/357, **f** 361/361, **g** 361/363, **h** 357/363, **i** 357/359

**Fig. 3:**
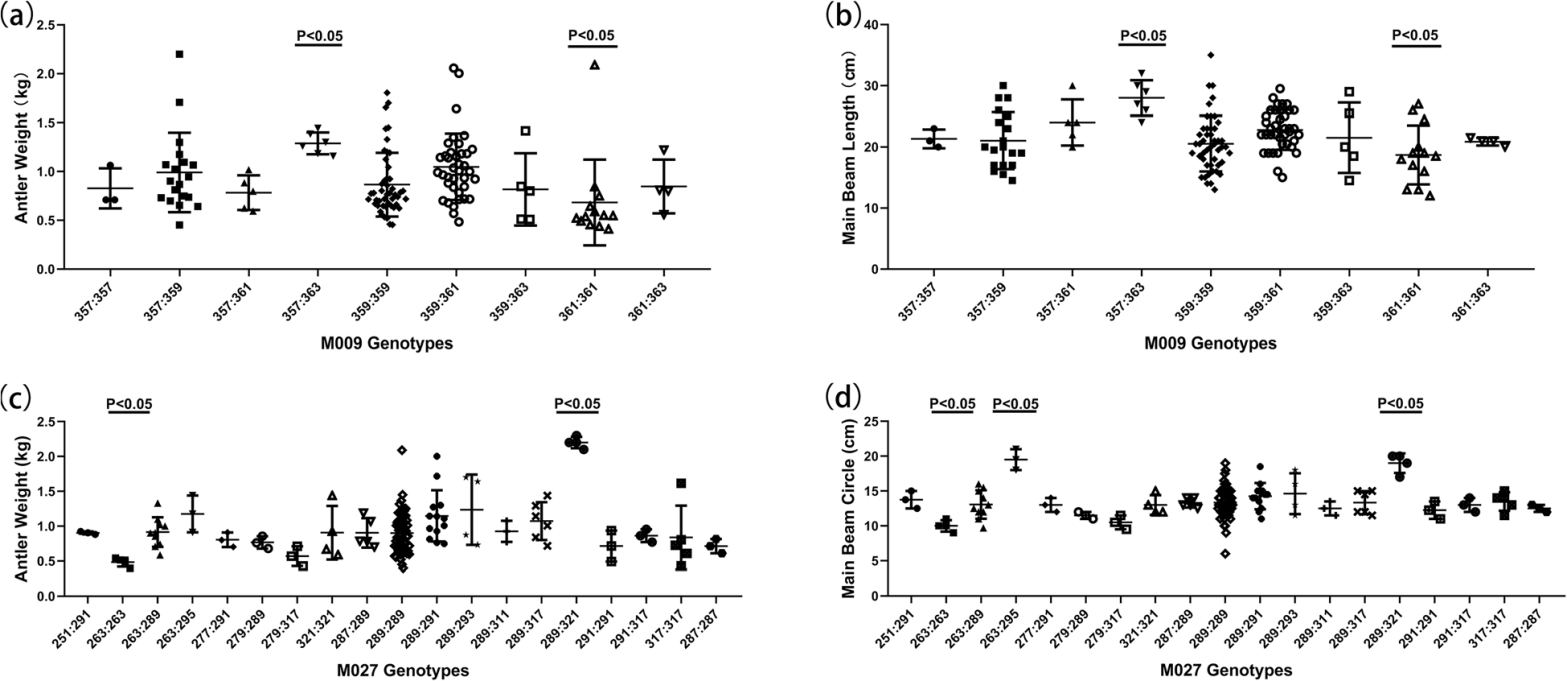
Correlation analysis between microsatellites and velvet antler traits of Sika deer. **a** Correlation analysis between M009 genotypes and velvet antler weight. **b** Correlation analysis between M009 genotypes and main beam length of velvet antler. **c** Correlation analysis between M027 genotypes and velvet antler weight. **d** Correlation analysis between M027 genotypes and main beam circle of velvet antler

Of particular concern was M009, which screened 9 genotypes of Sika deer (Fig. 2). Among them, genotype 357/363 had the highest mean value of velvet antler weight and main beam length. On the contrary, genotype 361/361 had the lowest mean values of velvet antler weight and main beam length (Table 5, Fig. 3a, b). The correlation between M009 alleles and velvet antler traits of Sika deer were further analyzed. The result showed that locus 363 and 357 had the highest mean value of velvet antler weight and main beam length, and there was no significant difference in contribution between them. However, locus 361 was the opposite (Table 6). Combined with other genotype individuals, it could be speculated that both the locus 357 and 363 of genotype 357/363 had a positive effect, while the locus 361 of genotype 361/361 had a negative impact on velvet antler weight and main beam length of two-branched velvet antler of shuangyang Sika deer.

**Table 5:** Correlation analysis between M009 and velvet antler traits of Sika deer

M009	Velvet Antler Weight (kg)	Main Beam Length (cm)
357:363	1.379 ± 0.028^a^	28.000 ± 0.583^a^
359:363	1.049 ± 0.033^ab^	22.803 ± 0.760^ab^
357:359	0.991 ± 0.041^ab^	21.026 ± 0.956^b^
359:359	0.866 ± 0.027^ab^	20.543 ± 0.708^b^
361:363	0.848 ± 0.045^ab^	20.825 ± 1.225^b^
357:357	0.828 ± 0.041^ab^	21.333 ± 1.185^b^
359:361	0.818 ± 0.045^ab^	21.500 ± 1.344^b^
357:361	0.784 ± 0.034^ab^	24.000 ± 1.143^ab^
361:361	0.523 ± 0.015^b^	15.954 ± 0.469^b^

In the same column, those with the same letters indicate that there was no significant difference between the two genotypes and those with different letters indicate that there was significant difference between the two genotypes

**Table 6:** Correlation analysis between M009 alleles and velvet antler traits of Sika deer

M009 Alleles	Velvet Antler Weight (kg)	Main Beam Length (cm)
363	1.073 ± 0.031^a^	23.288 ± 0.728^a^
357	1.000 ± 0.034^ab^	22.653 ± 0.839^ab^
359	0.924 ± 0.020^b^	21.183 ± 0.481^b^
361	0.652 ± 0.021^c^	18.140 ± 0.626^c^

In the same column, those with the same letters indicate that there was no significant difference between the two genotypes and those with different letters indicate that there was significant difference between the two genotypes

**Fig. 4:**
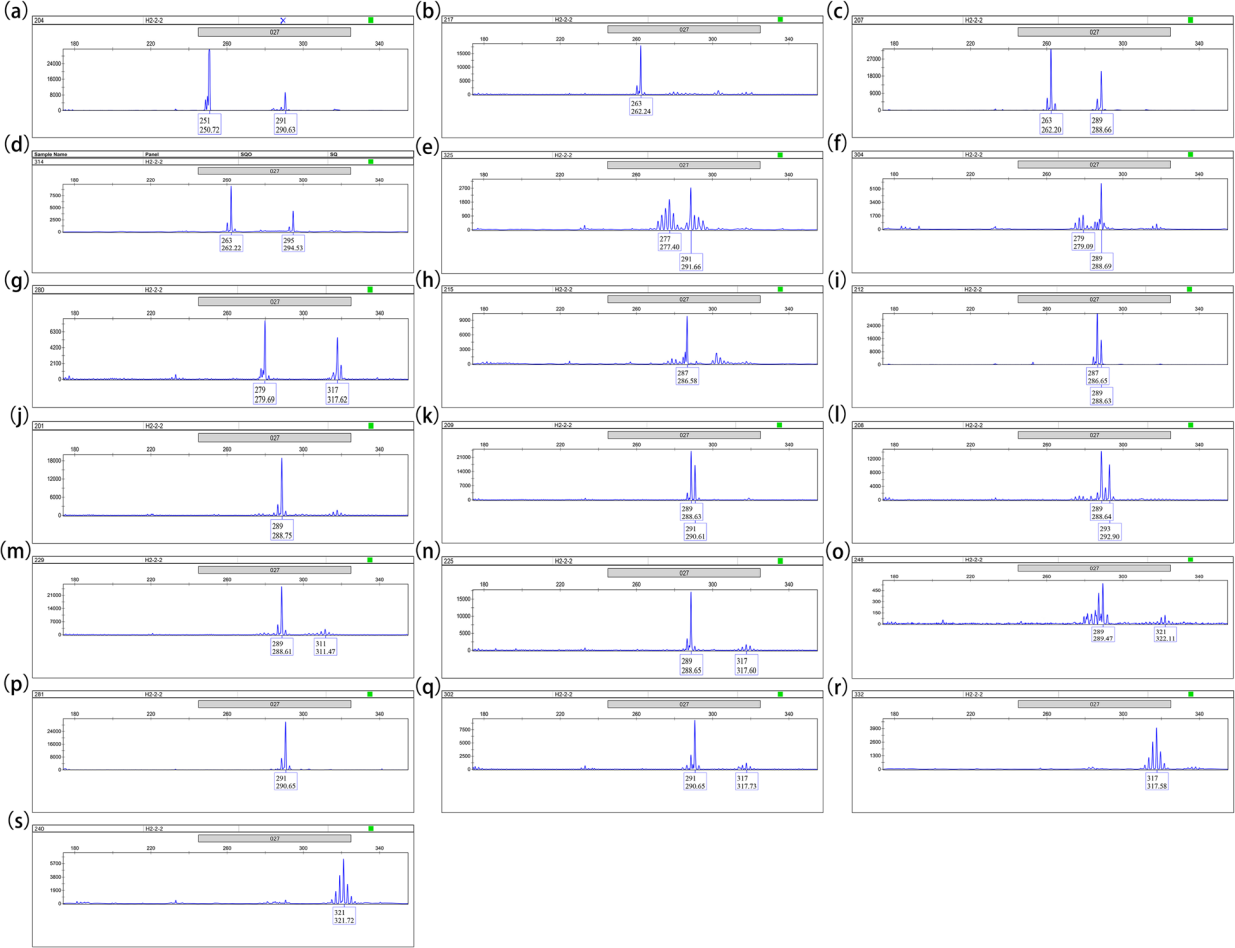
Typical fluorescence signals of the M027 microsatellite loci by capillary electrophoresis fragment analysis. The numbers above the sharp peaks represent length scales based on the internal size standard (bp). The numbers below the sharp peaks represent fragment lengths of PCR amplifications (bp). **a** 251/291, **b** 263/263, **c** 263/289, **d** 263/295, **e** 277/291, **f** 279/289, **g** 279/317, **h** 287/287, **i** 287/289, **j** 289/289, **k** 289/291, **l** 289/293, **m** 289/311, **n** 289/317, **o** 289/321, **p** 291/291, **q** 291/317, **r** 317/317, **s** 321/321

Another concern was M027, which screened 19 genotypes of Sika deer (Fig. 4). Among them, genotype 289/321 had the highest mean value of velvet antler weight and main beam circle. On the contrary, genotype 263/263 had the lowest mean values of velvet antler weight and main beam circle (Table 7, Fig. 3c, d). The correlation between M027 alleles and velvet antler traits of Sika deer were further analyzed. The result showed that locus 295 and 321 had the highest mean value of velvet antler weight and main beam circle. However, the contribution of locus 289 that we concerned was not significant. On the contrary, locus 263 and 279 had the lowest mean value of velvet antler weight and main beam circle (Table 8). Combined with other genotype individuals, it could be speculated that the locus 321 of genotype 289/321 had a positive effect, while the locus 263 of genotype 263/263 had a negative impact on velvet antler weight and main beam circle of two-branched velvet antler of shuangyang Sika deer.

**Table 7:** Correlation analysis between M027 and velvet antler traits of Sika deer

M027	Velvet Antler Weight (kg)	Main Beam Circle (cm)
289:321	2.200 ± 0.041^a^	19.000 ± 0.345^a^
289:293	1.237 ± 0.063^b^	14.667 ± 0.772^b^
263:295	1.176 ± 0.045^bc^	19.500 ± 0.813^a^
289:291	1.147 ± 0.038^bc^	14.273 ± 0.492^bc^
289:317	1.073 ± 0.037^bc^	13.375 ± 0.495^bcd^
289:311	0.926 ± 0.032^bc^	12.500 ± 0.446^bcd^
263:289	0.915 ± 0.027^bc^	13.071 ± 0.396^bcd^
321:321	0.906 ± 0.048^bc^	13.000 ± 0.722^bcd^
287:289	0.904 ± 0.032^bc^	13.300 ± 0.512^bcd^
251:291	0.903 ± 0.019^bc^	13.750 ± 0.275^bcd^
289:289	0.883 ± 0.021^bc^	13.333 ± 0.267^bcd^
291:317	0.864 ± 0.024^bc^	13.000 ± 0.351^bcd^
317:317	0.838 ± 0.047^bc^	13.500 ± 0.794^bcd^
277:291	0.805 ± 0.024^bc^	13.000 ± 0.394^bcd^
279:289	0.767 ± 0.022^bc^	11.500 ± 0.338^bcd^
291:291	0.715 ± 0.034^bc^	12.250 ± 0.613^bcd^
287:287	0.714 ± 0.022^bc^	12.500 ± 0.403^bcd^
279:317	0.570 ± 0.024^bc^	10.500 ± 0.457^cd^
263:263	0.485 ± 0.014^c^	10.000 ± 0.294^d^

In the same column, those with the same letters indicate that there was no significant difference between the two genotypes and those with different letters indicate that there was significant difference between the two genotypes

**Table 8:** Correlation analysis between M027 alleles and velvet antler traits of Sika deer

M027 Alleles	Velvet Antler Weight (kg)	Main Beam Circle (cm)
321	1.338 ± 0.065^a^	15.000 ± 0.789^a^
293	1.237 ± 0.069^ab^	14.625 ± 0.860^ab^
295	1.176 ± 0.054^abc^	19.500 ± 0.951^c^
289	0.966 ± 0.022^bc^	13.520 ± 0.314^bd^
291	0.962 ± 0.032^bc^	13.509 ± 0.466^bd^
311	0.926 ± 0.039^bc^	12.500 ± 0.532^bd^
251	0.903 ± 0.023^bc^	13.750 ± 0.335^bd^
317	0.869 ± 0.034^bc^	12.977 ± 0.540^bd^
277	0.805 ± 0.029^bc^	13.000 ± 0.491^bd^
287	0.800 ± 0.028^c^	12.864 ± 0.463^bd^
263	0.789 ± 0.031^d^	12.819 ± 0.534^bd^
279	0.669 ± 0.028^d^	11.000 ± 0.478^e^

In the same column, those with the same letters indicate that there was no significant difference between the two genotypes and those with different letters indicate that there was significant difference between the two genotypes

## Discussion

EST microsatellite were powerful molecular markers for analyzing population genetic diversity, molecular breeding and functions [27]. However, to date, few studies about EST microsatellite markers had been reported from Sika deer. Development of EST microsatellite markers based on transcriptome data was still an economic and efficient development strategy of DNA molecular markers. Our study was the first attempt to obtain transcriptional information of Sika deer for the EST microsatellite and to further examine the diversity and velvet antler yield characters. We successfully and efficiently isolated 182,295 EST microsatellite markers by IlluminaHiSeq™2500 sequencing technology. So far, we have obtained the largest number of Sika deer microsatellite databases to facilitate future research. The distribution of EST microsatellites with 1–6 bp repeat motif showed that, mono-nucleotide is the most abundant motif type, followed by di-nucleotide, tri-nucleotide, tetra-nucleotide, penta-nucleotide and hexa-nucleotide repeats. This was similar to the previous study on the development of microsatellites by reduced-representation genome sequencing in Sika deer [21]. The difference was that the number of microsatellites obtained by transcriptome sequencing was far more than that obtained by genome sequencing. However, the number of microsatellites highly repetitive motifs (≥10) from tri-nucleotide to hexa-nucleotide obtained by genome sequencing was more than that obtained by transcriptome sequencing. In this study, both mono-nucleotide and di-nucleotide repeats have been found to be the predominant repeat types. A/T, AC/GT, AGC/CTG were the most abundant repeat among mono, di and tri-nucleotide types, which were similar to previous studies by reduced-representation genome sequencing [21]. However, AATG/ATTC was the most frequent repeat among tetra-nucleotide types, unlike AAAC previously reported in Sika deer [21]. Thus, the EST microsatellite types obtained by transcriptome sequencing and reduced-representation genome sequencing without reference genomes were different. The microsatellite loci obtained from transcriptome showed lower polymorphism compared with genome but were more likely to be related to functional loci.

As we all know, Shuangyang Sika deer was the first breed that had been identified by the state in China. It had excellent traits such as high yield, strong adaptability, early maturity and so on. Among all the breeds, the breeding scale and social benefits of Shuangyang Sika deer were the largest. In order to further study the genetics of Shuangyang Sika deer and to screen microsatellites related to velvet antler growth characteristics, 29 highly polymorphic EST microsatellites were developed from 170 microsatellites expressed in velvet antler, which showed that transcriptome sequencing technology could be used to efficiently develop microsatellites. The indicators reflecting the genetic diversity of the population mainly include He, Ho, PIC, Ne and so on. Firstly, the Ho (Ho = 0.5127) of 29 loci developed from Shuangyang Sika deer was lower than He (He = 0.6018). The result showed that there was a certain degree of inbreeding in the Shuangyang Sika deer population, which was supposed to be caused by geographical isolation. Secondly, compared with previous studies, the PIC values of Shuangyang Sika deer (PIC = 0.545) was lower than Linyi Sika deer (PIC = 0.662), Yangzhou Sika deer (PIC = 0.570) and Jilin Sika deer (PIC = 0.680, Shuangyang was a city in Jilin Province). The result was mainly due to the relatively conservative coding region, the polymorphism of the EST microsatellite derived from the transcriptome was lower than that of the G microsatellite derived from the genome. Then it was caused by the difference in geographical location and breeding degree. Finally, the Na of some EST microsatellites, such as M027 (Na = 12, Ne = 2.1), M082 (Na = 14, Ne = 3.0) and M176 (Na = 10, Ne = 3.8), was much larger than the Ne. However, the average Na of 29 EST microsatellites was close to Ne (Na = 5.6, Ne = 2.6). This indicated that some of the microsatellite alleles were unevenly distributed in the population, but the overall distribution remains uniform. The reason for this phenomenon may be that during the long-term breeding process of Shuangyang Sika deer, some loci were subject to strong selection pressures associated with velvet antler growth characteristics, resulting in uneven distribution of alleles.

Multiple linear regression analysis identified that velvet antler weight was positively correlated with main beam circle, main beam length, mouth circle, mouth length, first tine circle. Main beam circle, main beam length, mouth circle, mouth length and first tine circle have the potential to predict 86.9% of the variability of velvet antler weight. Surprisingly, there was no linear relationship between first tine length and velvet antler weight. Therefore, the data of first tine length have not been further analyzed. One of most important implications of this study was that 8 EST microsatellites were found can be used in correlation analysis of velvet antler growth characteristics. In particular, M009 and M027 can be used as molecular markers in the breeding process of weight of the two-branched velvet antler. Velvet antler weight was very important for the production performance of Chinese Sika deer [28]. Therefore, it was necessary to develop large-scale molecular markers associated with velvet antler weight by sequencing. Hu et al. (2019) found that 94 SNP genetic variations were related to the three-branched velvet antler weight [28]. There were two significant differences between our study and that of Hu et al. (2019). First, Hu et al. (2019) chose the three-branched velvet antler (growth period of 75 days) as the research object, while we chose the two-branched velvet antler (growth period of 45 days) as the research object. Compared with the three-branched velvet antler, the two-branched velvet antler had lowered the rate of ossification, more organic components and greater medicinal value [29]. Secondly, most of the SNP sites developed by Hu et al. (2019) were in the intron region of the gene. However, the microsatellites we developed were in the exon region of the gene, which may obtain the concerned functional genes possibly associated with the growth and development of velvet antler. As we all know, complex characteristics could not be completely controlled by a single gene, and it required a series of genes. So far, screening weight related molecular markers of velvet antlers were still very limited. Molecular markers must be developed by using high-throughput omics data. In this study, microsatellite databases of Sika deer were developed by using transcriptome sequences. We investigated candidate EST microsatellite related to the characteristics in two-branched velvet antler. The result would facilitate further studies breeding of Sika deer and genetic mechanism of velvet antler weight difference.

## Conclusion

In this study, we conducted a transcriptome survey of sika deer using next-generation sequencing technology. We obtained useful data of EST microsatellite, such as the frequency and distribution. Secondly, EST microsatellites were selected and further characterized for polymorphism analysis. Finally, the marker-trait association was tested for important and kernel characteristics of velvet antler in sika deer. The development of a large number of sika deer molecular markers could help to breeding and culture.

## Materials

### Sample collection

Twelve male Sika deer (three for each of the four developmental stages) were exsanguinated after general anesthesia. Ten types of tissue (adrenal, velvet antler, brain, heart, kidney, lung, liver, skeletal muscle, spleen and testes) from one-year-old (juvenile), 3 years old (adolescence), 5 years old (adult), and 10 years old (aged) Sika deer were then collected. Fresh samples of these tissues were immediately frozen in liquid nitrogen, and then stored at − 80 °C for RNA extraction.

One hundred forty healthy 2-year-old male Shuangyang Sika deer were randomly selected. All Sika deer came from the commercial farm under the same living conditions (Jilin Province). The samples were blooded from jugular vein of Sika deer after general anesthesia. Anticoagulant blood was stored in − 20 °C until DNA extraction. The price of two-branched velvet antler was higher than that of three-branched velvet antler in Asian market. It took about 45 days for velvet antler to grow into two-branched velvet antler. Therefore, after individual anesthesia, velvet antlers were harvested for measuring (velvet antler weight, main beam length, main beam circle, first tine length, first tine circle, mouth length, mouth circle) and sample collection at 50 days of growth.

### Development of EST microsatellite markers derived from transcriptome of Sika deer

We have carried out Illumina Hiseq 2500 technology to perform high-throughput deep sequencing of the Sika deer transcriptome, with a cDNA library constructed by one RNA pool which had an equal quantity of total RNA extracted from adrenal, velvet antler, brain, heart, kidney, lung, liver, skeletal muscle, spleen and testes of Sika deer (Specific methods referred to the articles which we have published [3]. By comparing the professional software such as picard-tools and samtools, the duplicate reads were removed, and the original results were filtered. All types of microsatellite from mono-nucleotides to hexa-nucleotides were identified from the unigenes using MISA software under default parameter settings: a minimum of ten repeats for mono-nucleotide, six repeats for di-nucleotide microsatellites, five repeats for tri-nucleotide, tetra-nucleotide, penta-nucleotide and hexa-nucleotide microsatellites. Finally, in order to verify the identified microsatellite markers, all the microsatellites data need to be further screened. Polymorphism was one of the important criteria for judging the usability of microsatellite markers. The length of microsatellite fragment was one of the important factors which affecting its polymorphism. The polymorphism was high when the length of microsatellite was more than 20 bp, medium when the length of microsatellite was between 12 and 20 bp, and very low when the length of microsatellite was less than 12 bp [30]. Therefore, in this study, the length of less than 12 bp microsatellite was removed. Among the mono-nucleotide motifs, the most common motif was A/T. It should be noted that mono-nucleotide duplication was prone to mismatches, leading to sequencing failure, so this study was not an option. A total of 200 EST microsatellites expressed in velvet antler were selected as candidate molecular markers.

### EST microsatellites primer pair design

In this study, TP-M13- microsatellite PCR method was used to screen for primer [31]. Three primers were designed for PCR amplification: the first primer was F + M13, i.e. the 5’end of the EST microsatellite forward primer and M13 (5′-TGTAAAACGACGCCAGT-3′) to be linked together. The second primer was the EST microsatellite reverse primer, and the third primer was M13 primer fluorescently labeled at the 5’end with Cy5.

### DNA extraction, EST microsatellite markers validation and polymorphism examination

The total blood genomic DNA was extracted from 140 Sika deer following the traditional proteinase K and phenol-chloroform extraction method and verified by electrophoresis on 1% agarose gel. DNA was stored at − 20 °C until used for PCR amplification.

Fluorescence PCR amplification was carried out in a 20 μL volume containing 8 μL 10× Buffer I, 0.4 μL TP -M13(5 M), 2 μL specific primer(5 M), 0.2 μL Taq DNA Polymerase, 2 μL DNA, 7.6 μL ddH_2_O. Taq DNA Polymerase was purchased from Takara Co.,Ltd. The primers were synthesized from Beijing Microread Genetics Co.,Ltd. The PCR program consisted of an initial step of 95 °C for 5 min, followed by 30 cycles of 94 °C for 30 s, 56 °C for 45 s, and 72 °C for 45 s, followed by 10 cycles of 94 °C for 30 s, 53 °C for 45 s, 72 °C for 45 s, and a final extension at 72 °C for 12 min. The amplified products were evaluated on the ABI 3730XL DNA capillary sequencer with GeneScan 500 ROX Size Standard (Applied Biosystems, USA). The criterion for accepting a peak as polymorphic was that one peak was greater than or equal to one tenth of the other.

### Data analysis

GenAlEx version 6.5 was employed for the allele data processing, which included the number of expected heterozygosity (H_e_), observed heterozygosity (H_o_), polymorphism information content (PIC), number of alleles (N_a_), effective number of alleles (N_e_) [32]. GENEPOP software was used to investigate linkage disequilibrium and to determine deviation from Hardy–Weinberg equilibrium [33]. The correlation between velvet antler weight and main beam length, main beam circle, first tine length, first tine circle, mouth length, mouth circle of velvet antler of Shuangyang Sika deer was analyzed by multiple linear regressions. One-way analysis of variance (one-way ANOVA)and post hoc Bonferroni tests were performed on significant analysis of EST microsatellite markers with growth characteristics (velvet antler weight, main beam length, main beam circle, first tine length, first tine circle, mouth length, mouth circle) of 140 individuals in velvet antler. A *p* value≤0.05 was considered statistically significant difference.

## Supplementary information

**Additional file 1.** Distribution of EST microsatellite type in transcriptome of Sika deer.

## Data Availability

All the data generated in the present research is contained in this manuscript.

## Abbreviations

SNPs: Single nucleotide polymorphisms
RAPD: Random amplified polymorphic DNA
AFLPs: Amplified fragment length polymorphisms
SRAPs: Sequence-related amplified polymorphisms
EST microsatellite: Expressed sequence tag microsatellite
He: Expected heterozygosity
Ho: Observed heterozygosity
PIC: Polymorphism information content
Na: Number of alleles
Ne: Effective number of alleles
