# Supplementary material for: Development of novel EST microsatellite markers for genetic diversity analysis and correlation analysis of velvet antler growth characteristics in Sika deer
Source: Hereditas. 2020 Jun 26;157:24. doi: 10.1186/s41065-020-00137-x (PMC7320565; doi:10.1186/s41065-020-00137-x)
Supplement: Supplementary file 1 — Additional file 1. Distribution of EST microsatellite type in transcriptome of Sika deer. [file 41065_2020_137_MOESM1_ESM.docx]

Additional File 1. Distribution of EST microsatellite type in transcriptome of Sika deer

| Motif | Repeats | Percentage (%) | |  | Motif | Repeats | Percentage (%) |
| --- | --- | --- | --- | --- | --- | --- | --- |
| A/T | 121502 | - |  | | AAGT/ACTT | 10 | 0.02 |
| C/G | 6385 | - |  | | AATC/ATTG | 25 | 0.05 |
| Mono-nucleotide | 127887 | - |  | | AATG/ATTC | 250 | 0.46 |
| AC/GT | 26522 | 48.75 |  | | AATT/AATT | 18 | 0.03 |
| AG/CT | 7722 | 14.19 |  | | ACAG/CTGT | 86 | 0.16 |
| AT/AT | 4468 | 8.21 |  | | ACAT/ATGT | 61 | 0.11 |
| CG/CG | 546 | 1.00 |  | | ACCC/GGGT | 9 | 0.02 |
| Di-nucleotide | 39258 | 72.15 |  | | ACCG/CGGT | 2 | 0.00 |
| AAC/GTT | 1703 | 3.13 |  | | ACCT/AGGT | 9 | 0.02 |
| AAG/CTT | 554 | 1.02 |  | | ACGC/CGTG | 21 | 0.04 |
| AAT/ATT | 1182 | 2.17 |  | | ACGG/CCGT | 16 | 0.03 |
| ACC/GGT | 1051 | 1.93 |  | | ACTC/AGTG | 63 | 0.12 |
| ACG/CGT | 49 | 0.09 |  | | ACTG/AGTC | 23 | 0.04 |
| ACT/AGT | 113 | 0.21 |  | | AGAT/ATCT | 61 | 0.11 |
| AGC/CTG | 3420 | 6.29 |  | | AGCC/CTGG | 31 | 0.06 |
| AGG/CCT | 1665 | 3.06 |  | | AGCG/CGCT | 17 | 0.03 |
| ATC/ATG | 453 | 0.83 |  | | AGCT/AGCT | 2 | 0.00 |
| CCG/CGG | 3296 | 6.06 |  | | AGGC/CCTG | 48 | 0.09 |
| Tri-nucleotide | 13486 | 24.79 |  | | AGGG/CCCT | 64 | 0.12 |
| AAAC/GTTT | 191 | 0.35 |  | | ATCC/ATGG | 127 | 0.23 |
| AAAG/CTTT | 109 | 0.20 |  | | ATGC/ATGC | 14 | 0.03 |
| AAAT/ATTT | 189 | 0.35 |  | | CCCG/CGGG | 27 | 0.05 |
| AACC/GGTT | 35 | 0.06 |  | | CCGG/CCGG | 3 | 0.01 |
| AACT/AGTT | 8 | 0.01 |  | | Tetra-nucleotide | 1603 | 2.95 |
| AAGC/CTTG | 6 | 0.01 |  | | Penta-nucleotide | 56 | 0.10 |
| AAGG/CCTT | 78 | 0.14 |  | | Hexa-nucleotide | 5 | 0.01 |
